# Cloning and Functional Characterization of c-Jun NH_2_-Terminal Kinase from the Mediterranean Species of the Whitefly *Bemisia tabaci* Complex

**DOI:** 10.3390/ijms140713433

**Published:** 2013-06-27

**Authors:** Lan-Lan Wang, Huang Huang, Chang-Rong Zhang, Jun Xia, Shu-Sheng Liu, Xiao-Wei Wang

**Affiliations:** Ministry of Agriculture Key Laboratory of Agricultural Entomology, Institute of Insect Sciences, Zhejiang University, Hangzhou 310058, China; E-Mails: wanglanlan19850228@163.com (L.-L.W.); 18868106337@126.com (H.H.); zhangchangrong2006@163.com (C.-R.Z.); ptxiajun@gmail.com (J.X.); shshliu@zju.edu.cn (S.-S.L.)

**Keywords:** *Bemisia tabaci*, c-Jun amino-terminal kinase, fungal infection, mitogen-activated protein kinase, whitefly

## Abstract

c-Jun NH_2_-terminal kinase (JNK) signaling is a highly conserved pathway that controls gene transcription in response to a wide variety of biological and environmental stresses. In this study, a JNK from the invasive Mediterranean (MED) species of the whitefly *Bemisia tabaci* complex was cloned and characterized. The full-length JNK cDNA of MED consists of 1565 bp, with an 1176 bp open reading frame encoding 392 amino acids. Comparison of JNK amino acid sequences among different species showed that the sequences of JNKs are highly conserved. To reveal its biological function, the gene expression and functional activation of JNK were analyzed during various stress conditions. Quantitative RT-PCR analysis showed that the relative expression level of JNK remained hardly unchanged when the insects were transferred from cotton (a suitable host plant) to tobacco (an unsuitable host plant), infected with bacteria and treated with high and low temperatures. However, the mRNA level of JNK significantly increased when treated with fungal pathogens. Furthermore, we found that the amount of phosphorylated JNK increased significantly after fungal infection, while there is no obvious change for phosphorylated p38 and ERK. Our results indicate that the whitefly JNK plays an important role in whitefly’s immune responses to fungal infection.

## 1. Introduction

The whitefly *Bemisia tabaci* (Gennadius) (Hemiptera: Aleyrodidae) is a cryptic species complex composed of >35 morphologically indistinguishable species [1–4]. Some members of this species complex are considered a major pest for a range of agricultural, horticultural and ornamental crops causing damage directly through feeding and, indirectly, through transmission of over 100 plant viruses, primarily *Begomoviruses* [5–7]. In this cryptic species complex, the Middle East-Asia Minor 1 (MEAM1, commonly known as biotype B) and the Mediterranean (MED, known as biotype Q) are highly invasive and have colonized large areas worldwide [2,3,8]. The first major global invasion of MEAM1 commenced sometime in the late 1980s principally via the trade in ornamentals from its origins in the Middle East-Asia Minor region to at least 54 countries [2,7,9]. In recent years this has been followed by the global spread of MED from its origin in the countries bordering the Mediterranean Basin [2,4]. The global invasion of the two *B. tabaci* species is associated with their high capacity of surviving under various stresses [7,10,11]. However, the molecular mechanisms underlying their remarkable adaptability are still largely unknown.

Mitogen-activated protein kinase (MAPK) cascade is one of the most ancient and evolutionarily conserved signaling pathways that control a vast array of physiological processes [12]. Multicellular organisms have three well characterized subfamilies of MAPKs, the extracellular signal regulated kinase (ERK), the c-Jun NH_2_-terminal kinase (JNK) and the p38 [13]. Each plays a major role in regulation of intracellular metabolism and gene expression in many life activities including growth and development, disease, apoptosis and cellular responses to external stresses [14]. ERKs are involved in the regulation of meiosis, mitosis and post mitotic functions in differentiated cells, while JNK and p38 are critical for the stress-related signal transduction pathways conveying signals from the cell surface into the nucleus to initiate gene expression [15,16]. Previous studies found that JNK may be involved in cellular response to environmental stresses [17–19] and immune defense to virus and bacteria [16,20,21].

In insects, the roles of MAPK signaling pathway have been demonstrated in many aspects. For example, Fujiwara and Denlinger [22] have demonstrated that p38 MAPK is a likely component of the signal transduction pathway triggering rapid cold hardening in the flesh fly *Sarcophaga crassipalpis.* Their experiment on *Bombyx mori* suggested the potential role of MAPK in terminating the embryonic diapause [23]. In addition, MAPK is a component of the insect immune system that is activated in response to bacterial and fungal infection [24–27]. Mizutani *et al*. [20] had demonstrated that the JNK of mosquito was activated within 15 min by stimulation with heat-killed bacteria, and this quick activation reached a peak at 30 min. Moreover, Chen *et al*. [27] demonstrated that the p38 pathway-mediated stress response contributes to *Drosophila* host defense against bacterial and fungal infection. Previously, we cloned p38 and ERK genes from the MED species of the *B. tabaci* complex, and demonstrated that p38 pathway is important during whiteflies’ response to low temperature stress [28]. However, until now, information about the function of *B. tabaci* MAPKs under other stress conditions is still lacking.

To gain further insights into the characteristics of MAPK and their functional roles in *B. tabaci*, we cloned the JNK gene of the MED *B. tabaci* and examined the mRNA levels of MAPKs in different developmental stages of the whitefly. Then, we investigated the mRNA level of MAPKs in whiteflies under various stress conditions including heat, cold, and shift of host plants. Finally, we analyzed the activation of ERK, p38 and JNK in whiteflies treated with fungi and bacteria.

## 2. Results

### 2.1. Cloning and Sequence Analyses of JNK

**Figure 1:**
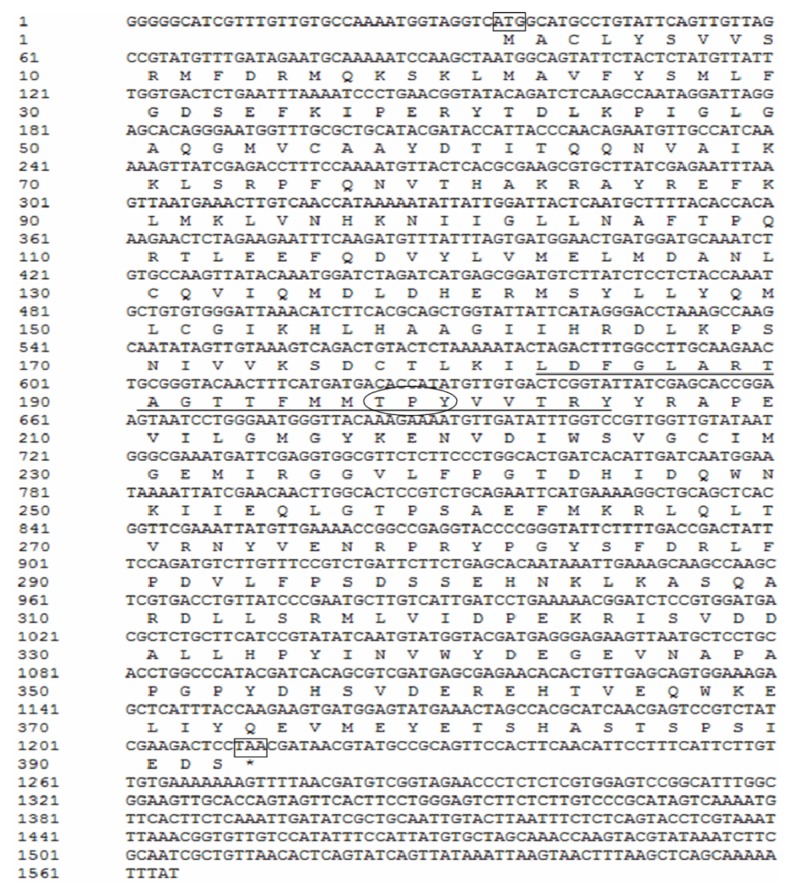
Nucleotide and predicted amino acid sequences of c-Jun NH_2_-terminal kinase (JNK) from the Mediterranean species of whitefly *Bemisia tabaci* complex. The start codon (ATG) and stop codon (TAG) are boxed. The active loop is underline. The conserved motif (TPY) is circled.

The full-length cDNA of MED *B. tabaci* JNK (GenBank Accession number JF905627) consists of 1565 bp, with an 1176 bp open reading frame (ORF) which encodes 392 amino acids. The JNK cDNA sequence contains a 5′ untranslated region (UTR) of 32 nucleotides and a long 3′ UTR of 356 nucleotides. The calculated molecular mass of JNK is 45.14 kDa with estimated isoelectric point of 5.97. The amino acid sequence of *B. tabaci* JNK is 88% identical to JNK of *Aedes albopictus* and 90% to that of *Drosophila*. The predicted whitefly JNK protein contains the active site, ATP binding site, substrate site and active loop (amino acid: 182–204) with a specific TPY motif (Figure 1).

### 2.2. Homology and Phylogenetic Analyses of *Bemisia tabaci* JNK

**Figure 2:**
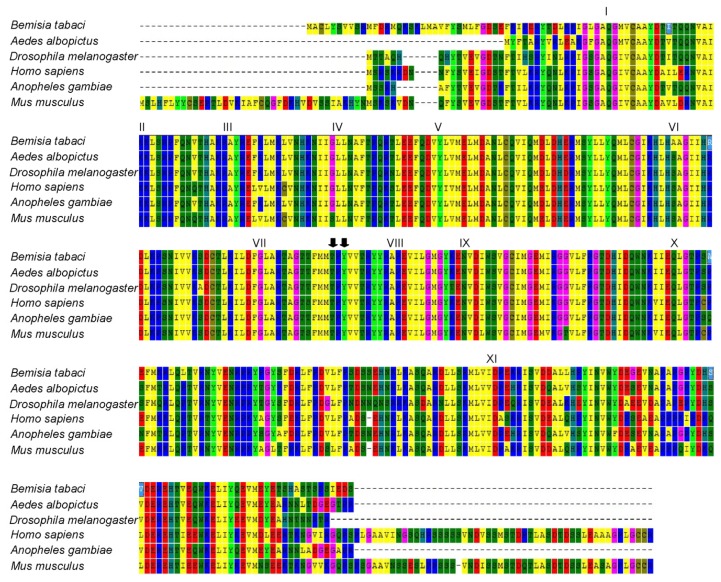
Multiple sequence alignments of JNKs from a variety of species. The amino acid sequence of *Bemisia tabaci* JNK was compared with those of other species. Black arrow indicates the phosphorylation sites on Thr and Tyr. The kinase subdomains are marked with roman numerals above the sequences.

Previous studies have demonstrated that JNK activation is mediated by the dual phosphorylation of the Thr-Pro-Tyr (TPY) sequence on Thr and Tyr [29]. Comparison of amino acid sequences among different species showed that the sequences of JNKs are highly conserved. MED *Bemisia tabaci* JNK contains all the conserved residues of protein kinases with subdomains I–XI. The dual phosphorylation motif (TPY), required for JNK activation is located between kinase subdomains VII and VIII (Figure 2). Overall, the amino acid sequence comparison with other species indicated that this sequence is the JNK in MED *B. tabaci.*

**Figure 3:**
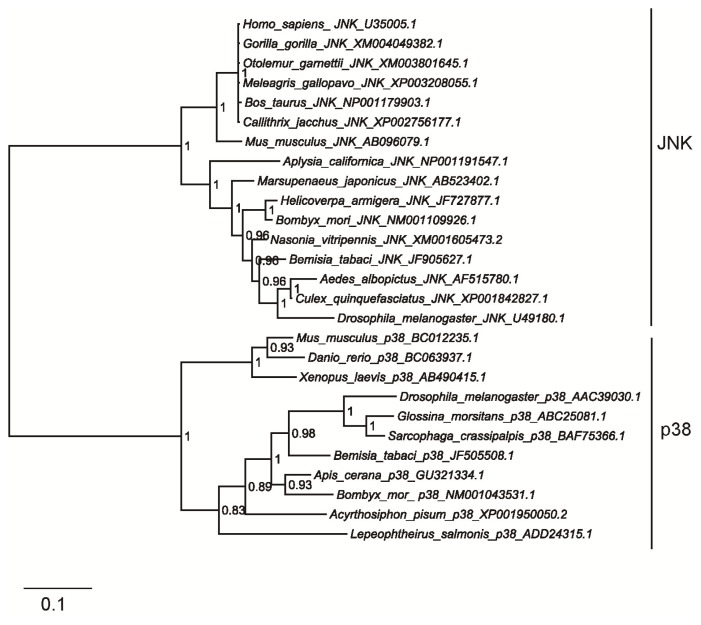
Phylogenetic analysis of JNKs from different species. A phylogenetic analysis was conducted based on a multiple alignment of JNK and p38 amino acid sequences retrieved from the GenBank database. Numbers at the nodes are posterior probability (10,000,000 generations). The scale bar is 0.1.

Phylogenetic tree was constructed based on the amino acid sequences of selected JNK protein sequences by the Bayesian analysis [30] using MrBayes 3.1. All the JNK clustered together as a subgroup and p38 clustered to another group (Figure 3). The phylogenetic analysis indicated that whitefly JNK are clustered and formed a sister group with JNK genes from other insect species. The topology tree approximately reflected the taxonomic classification of the corresponding species.

### 2.3. Expression in Different Developmental Stages

**Figure 4:**
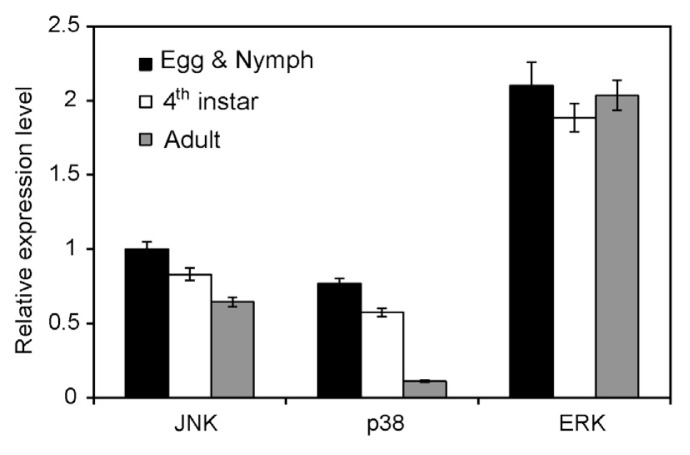
Quantification of JNK gene expression at different developmental stages. Each symbol and vertical bar represents the mean ± SD (*n* = 3).

To understand the physiological roles of MAPKs in the whitefly, the relative expression levels of JNK, p38 and ERK were detected by qRT-PCR at different developmental stages of *B. tabaci*. The results showed that the expression levels of ERK were consistent at different developmental stages and were significantly higher than that of JNK and p38. For the expression of JNK, there was a slight decrease with the development of the whitefly. However, the mRNA levels of p38 were significantly lower in adults than in egg and nymphs or 4th instars (Figure 4).

### 2.4. Effect of Host Shift on MAPK Activation

**Figure 5:**
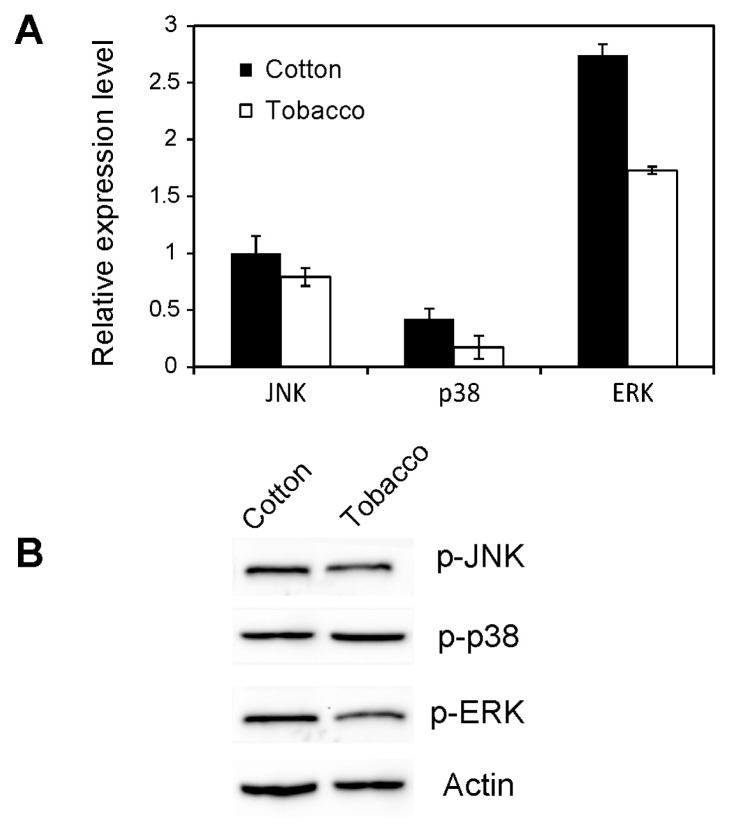
Effects of host shift on JNK, p38 and extracellular signal regulated kinase (ERK) in whiteflies. (**A**) Effect of host shift on mRNA level; (**B**) Effect of host shift on the phosphorylation level of JNK, p38 and ERK. Actin was used as a protein loading control. Each symbol and bar represents the mean ± SD (*n* = 3).

To reveal the function of MAPKs in MED *B. tabaci*, the gene expression levels of JNK, p38 and ERK were measured under various stress conditions. When whitefly adults were transferred from cotton (a favorable host plant) to tobacco (an unfavorable host plant) for 24 h, the relative expression level of ERK significantly decreased, while the level of JNK and p38 remained nearly unchanged (Figure 5A). Next, we analyzed the activation of MAPK in whiteflies on different host plants by immunoblotting with antiphospho-p38, -ERK, and -JNK antibodies. As the phosphorylation sites are highly conserved, commercial anti-phospho MAPK antibodies from *Drosophila* can be used to detect phosphorylated MAPKs in MED *B. tabaci*. As shown in Figure 5B, the phosphorylation of p38, ERK and JNK had no significant change on cotton and tobacco plants.

### 2.5. Effect of *Pseudomonas aeruginosa* on MAPK Activation

**Figure 6:**
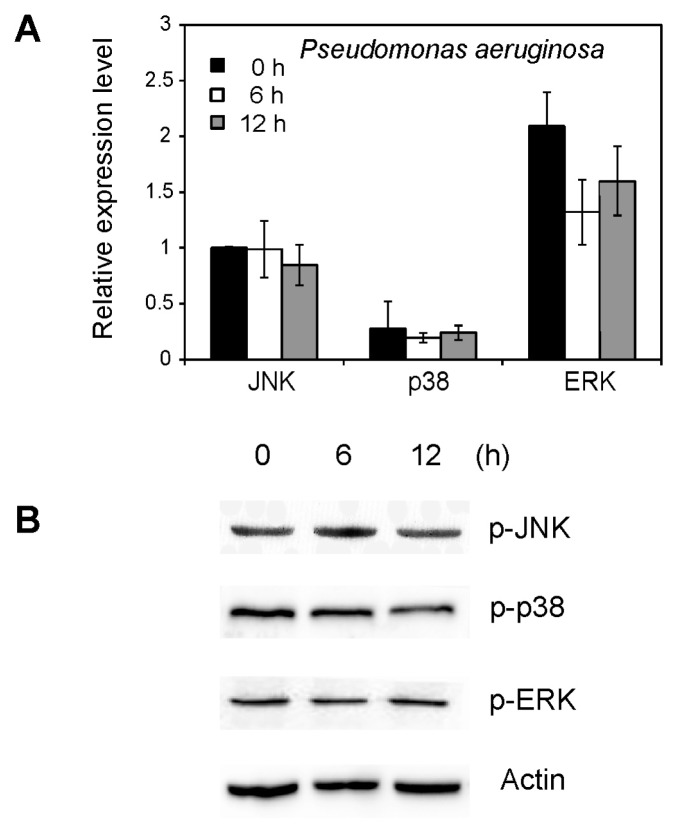
Effects of bacterial treatment on MAPK. (**A**) qRT-PCR analysis of JNK, p38 and ERK gene expression in whiteflies treated with bacterial for 0, 6 and 12 h; (**B**) Phosphorylation level of JNK, p38 and ERK in whiteflies analyzed by western blot. Each symbol and vertical bar represents the mean ± SD (*n* = 3).

To elucidate the role of MED *B. tabaci* MAPK in immune response, the effect of bacterial treatments on the gene expression and activation of JNK, p38 and ERK was examined. At both time points, MAPK in the whiteflies treated with *P. aeruginosa* showed no significant difference from those treated with Tween 20 (control) (Figure 6A). Similarly, the protein phosphorylation levels of ERK, p38 and JNK were not elevated by bacterial treatment (Figure 6B).

### 2.6. MAPK Activation in Whiteflies Treated with *Beauveria bassiana*

**Figure 7:**
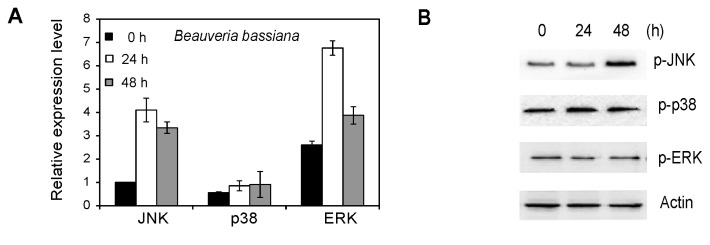
Effects of fungal treatment on MAPK. (**A**) qRT-PCR analysis of JNK, p38 and ERK in whiteflies treated with fungal for 0, 24 and 48 h; (**B**) Phosphorylation of JNK, p38 and ERK in whiteflies treated with fungal for 0, 24, 48 h. Each symbol and vertical bar represents the mean ± SD (*n* = 3).

Finally, the roles of whitefly MAPK in response to fungal infection were investigated. Our results showed that the mRNA level of JNK and ERK significantly increased when treated with *B. bassiana* for 24 h, while there is no significant change in p38 (Figure 7A). To further confirm that MAPKs are involved in the anti-fungus responses in whiteflies, we analyzed the activation of MAPK in whiteflies by immunoblotting with antiphospho-p38, -ERK, and -JNK antibodies. As shown in Figure 7B, an increase of phosphorylated JNK was observed at 48 h, while no change was detected for the activation of ERK and p38.

## 3. Discussion

MAPK is well known for its role in transmitting stress signals, such as environmental stresses, cold stress, and viral and bacterial infections [20,23,28,31–33]. Our previous studies have reported the roles of p38 MAPK in response to low temperature stress in MED *B. tabaci*. Here we cloned JNK from MED *B. tabaci* (Figure 1) and characterized the functions of whitefly MAPKs under various stress stimuli. Amino acid sequence analysis revealed that MED *B. tabaci* JNK had high homology with those of *A. albopictus*, *Drosophila* and human (Figures 2 and 3). It suggests that JNK signaling cascade systems may have similar roles in *B. tabaci*, such as cellular response to environmental stress [17,18] and immune response to pathogen infections [20,21]. Similarly, JNK has the phosphorylation motif of Thr-Pro-Tyr for activation as in mammals and *Drosophila* [25,34]. Therefore anti-phospho-JNK antibodies from other species can be used to detect phosphorylated JNK in *B. tabaci*.

Host plant can significantly influence the growth and condition of insects [19,35]. Previous experiments have examined the fecundity and longevity of adult whiteflies that were reared on cotton from egg to adult and transferred onto tobacco plants [36,37]. Whiteflies had a higher level of survival rate on cotton than on tobacco plants [36]. In addition, the longevity of the adult whiteflies reached on average 25 days on cotton, which were significantly longer than those on tobacco [37]. Therefore, host shift might affect the individual performance of insects. It seems that differential host adaptability may influence the capacity for an invader to compete with its indigenous competitors. MAPK activation has often been assumed to play an important role in whiteflies to adapt to new host plants, which was associated with their high potential capacity of survival under various environmental stresses. But, in our study, the MAPK was not activated when whiteflies was transferred from cotton to tobacco, suggesting that MAPK may not play significant role during the adaptation of *B. tabaci* to new host plants.

It has been known for more than a century that flies are strongly resistant to microbial infections [38]. A previous study has demonstrated that the p38 pathway-mediated stress response contributes to *Drosophila* host defense against pathogenic bacteria and fungi [27]. The *Drosophila* JNK is a component of the insect immune system that is activated in response to bacterial infection [25]. We investigated whether the JNK has a similar function in MED *B. tabaci*. Our results showed that JNK has little or no involvement in the response to bacterial infection. However, the mRNA and phosphorylated level of JNK in MED *B. tabaci* treated with fungal infection was significantly increased, indicating the important role of JNK signaling pathway in the interaction between fungi and whiteflies. Our results showed that the phosphorylation of JNK was not distinct at the first 24 h while it was significantly increased at 48 h. For fungal conidia to initiate infection, they must come into contact with, germinate on, and then penetrate the whitefly cuticle [39]. JNK signaling has been linked to stress responses, cell migration, apoptosis and immune responses in insects [12,20,21,25,40]. The study of signal transduction pathways in virulent fungi is especially important in view of their putative role in the regulation of pathogenicity [41]. Further studies to determine the functional roles of JNK signaling pathway during fungus–whitefly interactions are required.

## 4. Experimental Section

### 4.1. Source and Maintenance of Insects, Plants

The MED species of the *B. tabaci* complex (mtCOI GenBank Accession no. GQ371165) was used in this study. Whiteflies were reared on cotton plants (*Gossypium hirsutum* cv. Zhe-Mian 1793) in cages held in an insectary at temperatures of 25 to 27 °C, 60% relative humidity and 14 h light/10 h darkness. Tobacco (*Nicotiana tabacum* cv. NC89), an unsuitable host plant for the whitefly [36,37] was also used in this study. All plants were grown in insect-proof cages in greenhouse. Cotton and tobacco plants were obtained from seed sown directly into 15 cm pots and were cultivated to 7–8 true leaf stage for experiment.

### 4.2. Cloning of MED *B. tabaci* JNK

Initially, partial JNK cDNA sequence was obtained from the whitefly transcriptome deposited in NCBI (GenBank Accession no. EZ959222.1) [42]. 5′- and 3′-RACE PCR was performed to obtain the full length JNK gene using the SMART RACE cDNA Amplification Kit (Clontech, Palo Alto, CA, USA) following the manufacturer’s instructions. The gene-specific primers for the 5′ and 3′ RACE were as follows: 5′-TAATACCAGCTGCGTGAAGATGTT-3′ and 5′-TGGCAACATTCTGTTGGGTA-3′. The PCR product was cloned into the pMD18-T vector (Takara, Dalian, China) and sequenced at the GenScript Corporation (Nanjing, China). The cDNA sequences of JNK were submitted to the NCBI GenBank under the Accession number: JF905627.

### 4.3. Sequence and Phylogenetic Analysis

The nucleotide and deduced amino acid sequences of whitefly JNK were analyzed using software DNAMAN version 6 (http://www.lynnon.com) [43]. Theoretical isoelectric point values and molecular masses were calculated using Prot-Param tools (http://kr.expasy.org/tools/protparam.html) [44]. The sequence similarity of JNK from different species was analyzed and compared using the BLAST search programs (http://www.blast.ncbi.nlm.nih.gov/Blast.cgi) [45]. We used Clustal W (http://www.ebi.ac.uk/Tools/msa/clustalw2/) [46] to compare amino acid sequence of JNKs from the whitefly and other species. Phylogenetic relationship among the sequences was determined by reconstructing a protein phylogeny using MrBayes 3.1 (http://mrbayes.net) [47]. Nodes are posterior probability (10,000,000 generations).

### 4.4. RNA Isolation and Quantitative Reverse Transcription PCR (qRT-PCR) Analysis

The relative expression of genes was measured using qRT-PCR. Total RNA was isolated using the SV total RNA isolation system (Promega, Madison, VA, USA) according to the manufacturer’s protocol. RNA integrity was confirmed using the Nanodrop 2000C (Thermo, Minneapolis, MN, USA). cDNA was synthesized using the SYBR PrimeScript reverse transcription-PCR (RT-PCR) kit II (Takara, Dalian, China). Specific primers were designed as shown in Table 1. As an endogenous control, the expression of transcription initiation factor TFIID subunit (TAF) was measured in parallel. qRT-PCRs were carried out on the CFX96TM Real-Time system (Bio-Rad, Hercules, CA, USA) with SYBR green detection. Each gene was analyzed in triplicate and the average threshold cycle (*CT*) was calculated per sample. The expression level of target gene relative to TAF gene was determined based on the 2^−ΔΔCT^ method. Data shown are the mean of three independent reactions performed for each sample.

### 4.5. Western Blotting

About 100 insects were used for each sample. Whole whitefly bodies were homogenized in 100 μL of loading buffer (50 mM Tris-HCl, pH 6.8, 10% glycerol, 0.1% bromophenol blue, 1% β-mercaptoethanol and 2% SDS), boiled for 5 min, and 10 μL of each sample was applied to each lane. For Western blotting, protein samples were separated by 12% sodium dodecyl sulfate polyacrylamide gel electrophoresis (SDS-PAGE) and were then transferred to polyvinylidine difluoride membranes (Bio-Rad, Hercules, CA, USA). The membranes were blocked with 5% nonfat dry milk dissolved in PBS-Tween 20 (PBST) and then incubated with anti-phospho-p38, anti-phospho-ERK and anti-phospho-JNK (Cell Signaling Technology, Bevery, MA, USA). Secondary peroxidase-conjugated antibodies (MultiSciences Biotech, Hangzhou, China) were then added to the membrane and the signal was visualized using the ECL Plus Detection System (MultiSciences Biotech, Hangzhou, China). Equal loading of proteins was confirmed by use of anti-actin antibodies. At least three independent experiments were carried out to assess the phosphorylation level of each MAPK after the challenge of whitefly with different stimuli. In all cases the general trends were reproducible and the most representative western blot result was selected for presentation.

### 4.6. Effect of Host Shift on MAPK Activation

To examine the effect of host shift on MAPK activation, whiteflies collected from cotton plants (a suitable host plant) were transferred to tobacco plants (an unsuitable host plant) and new cotton plants (control). After 24 h, approximately 200 whiteflies were collected from tobacco and new cotton plants, respectively. By using whiteflies transferred from cotton to cotton as control, the side effects of whitefly transfer can be excluded. The whiteflies were then treated with liquid nitrogen and stored at −80 °C. Each treatment was replicated three times.

### 4.7. Bacterial Treatment

Adult whiteflies were fed with a solution of bacterial (*P. aeruginosa*) (1 × 108 CFU/mL) mixed with 10% sucrose solution through a double layer of Parafilm in 25-mm-diameter cylindrical containers. About 100 adult whiteflies were put in each container for bacteria feeding. Then, whiteflies were collected after feeding for 6 and 12 h respectively. Whiteflies fed with 10% sucrose solution only were used as control group. All treatments were replicated three times.

### 4.8. Fungal Treatment

The fungal strain *B. bassiana* ARSEF2860 from the RW Holley Center for Agriculture and Health (Ithaca, NY, USA) was preserved at 4 °C on slants of Sabouraud dextrose agar. Conidia harvested from cultures grown for 7 days at 27 °C and 95% relative humidity were suspended in 0.05% Tween 80 (10^10^ conidia/mL). Approximately 100 whitefly adults on a cotton leaf were placed in a Petri dish and exposed to a spray of 2 mL of the conidial suspension in an Automatic Potter Spray Tower (Burkhard Scientific Ltd., Middlesex, UK) as described previously [48]. The treated whiteflies were reared *in situ* at the regime of 27 °C and 95% RH and the number of living insects was recorded daily. Cadavers were moved to another moistened dish for incubation to verify whether they died of *B. bassiana* infection or not. For the control group, the same volume of 0.05% Tween 80 was sprayed onto the insects, followed by rearing under the same conditions.

## 5. Conclusions

In conclusion, we cloned the full-length JNK from the MED *B. tabaci* species and investigated the function of MAPKs in response to various stresses. These data highlights that while MAPKs are not important in mediating anti-bacteria response, JNK pathway is probably of great importance in response to fungal infection. However, future studies will be necessary to determine how the JNK pathway reacts after fungal infection and whether or not it is involved in the interaction between begomoviruses and whiteflies.

## Figures and Tables

**Table 1:** List of qRT-PCR primers used.

Gene	Primer sequence
JNK	forward primer: 5′-TGTTGAGCAGTGGAAAGAGC-3′
	reverse primer: 5′-TTCGATAGACGGACTCGTTG-3′

p38	forward primer: 5′-CATGGAAATTCTTGGAACCC-3′
	reverse primer: 5′-TTGGCTCCTTTGAACACTTG-3′

ERK	forward primer: 5′-TGGAATGGTCGTATCTGCAT-3′
	reverse primer: 5′-CATGCCTGAATCTTGTGAGG-3′

TAF	forward primer: 5′-TGTGGGACACCCATTATCAG-3′
	reverse primer: 5′-TGTGCAGCCAAGGAAATAAG-3′
